# Preoperative Administration of Amphotericin B in Orbital Mucormycosis Management: A Case Report

**DOI:** 10.1055/a-2558-6468

**Published:** 2025-04-11

**Authors:** Russel T. Wagner, Jacopo Berardinelli, Melanie B. Fukui, Sammy Khalili, Neil S. Mundi, Amin B. Kassam, Stephen J. Winkler

**Affiliations:** 1 Intent Medical Group, Endeavor Health Advanced Neurosciences Center, Northwest Community Hospital, Arlington Heights, Illinois, United States; 2 Division of Neurosurgery, Department of Neuroscience, Reproductive and Odontostomatological Sciences, University of Naples Federico II, Napoli, Campania, Italy

**Keywords:** orbital mucormycosis, amphotericin B, globe preservation, fungal infection, diabetes mellitus, preoperative management, intracranial extension, TRAMB injections, antifungal therapy, minimally invasive treatment

## Abstract

This case report presents a 29-year-old male with diabetes mellitus who developed rhino-orbito-cerebral mucormycosis (ROCM) that was successfully treated with liposomal amphotericin B orbital injections. Despite emergent endoscopic debridement, the patient's disease progressed intracranially and intraorbitally, but he declined further surgical intervention. Subsequently, due to rapid acute vision loss, we initiated transcutaneous retrobulbar amphotericin B (TRAMB) injections. Following these injections, visual acuity, motility, and intraorbital fungal burden improved despite intracranial progression. This report highlights the benefits of TRAMB administration in aggressive fungal infections and explores the mechanisms behind its effectiveness, particularly in globe preservation. By targeting the infection in an area with a relatively robust blood supply, TRAMB reduces surgical difficulty and improves overall outcomes.

## Introduction

Mucormycosis is a rare but aggressive opportunistic fungal infection caused by fungi of the order Mucorales, commonly found in soil, decaying matter, and food. 1 2 This life-threatening infection primarily affects immunocompromised individuals, often those with poorly controlled diabetes mellitus (DM). Commonly, rhino-orbito-cerebral mucormycosis (ROCM) presents in patients with diabetic ketoacidosis (DKA) 3 and is attributed to the altered metabolic state of hyperglycemia and acidosis. These conditions create a favorable environment for fungal proliferation by increasing free iron levels that enhance fungal growth. 4

The clinical presentation of ROCM typically begins with local symptoms such as facial pain, followed by rapid progression to systemic manifestations like fever, headache, and altered mental status. 3 4 5 Prompt treatment is critical to prevent mortality, as the angioinvasive nature of the infection can lead to tissue necrosis and rapid local spread. 2 Traditionally, standard management of ROCM has involved systemic antifungal therapy (typically amphotericin B) along with aggressive surgical debridement, which usually requires orbital exenteration. 2 3 4 5 6 7 8 9 10

During the COVID-19 pandemic in India, the surge of ROCM cases necessitated alternative treatments due to systemic liposomal amphotericin B becoming scarce and surgery often being delayed due to unavailable services. 2 7 11 Transcutaneous retrobulbar amphotericin B (TRAMB) injections emerged as a minimally invasive, globe-sparing intervention to address the rapid progression of orbital mucormycosis. Multiple studies, including those by Sharifi et al., Ramamurthy et al., Pathak et al., Singh et al., Ashraf et al., Sen et al., and Kaur et al., have demonstrated the success of TRAMB in halting disease progression and preserving vision in patients with early-stage orbital involvement 2 7 8 9 10 11 12 as well as offering an alternative to disfiguring surgeries like orbital exenteration. The rapid adoption and success of TRAMB in India during the pandemic highlighted its potential as a valuable adjunct to systemic antifungals and surgical debridement in managing COVID-19-associated ROCM.

Given the demonstrated efficacy of TRAMB in reducing the need for orbital exenteration, this case further explores the role of preoperative TRAMB injections in stabilizing ROCM and facilitating safer, more controlled surgical interventions. By drawing on this established research, we propose that preoperative administration of TRAMB may offer a paradigm shift in the management of this aggressive fungal infection, allowing clinicians to preserve ocular function while minimizing invasive procedures. 2 7 8 9 10 11 12

This case highlights the effectiveness of locally administered liposomal amphotericin B in controlling orbital mucormycosis, even with advanced spread. Its lipid-based formulation enhances drug delivery by offering a higher dose for a longer period of time to infected tissues while decreasing nephrotoxicity, 13 14 making it valuable for preoperative management. This approach offers targeted control of local disease, reducing the need for more invasive treatments while minimizing systemic side effects. 13 14

## Case Presentation

**Fig. 1:**
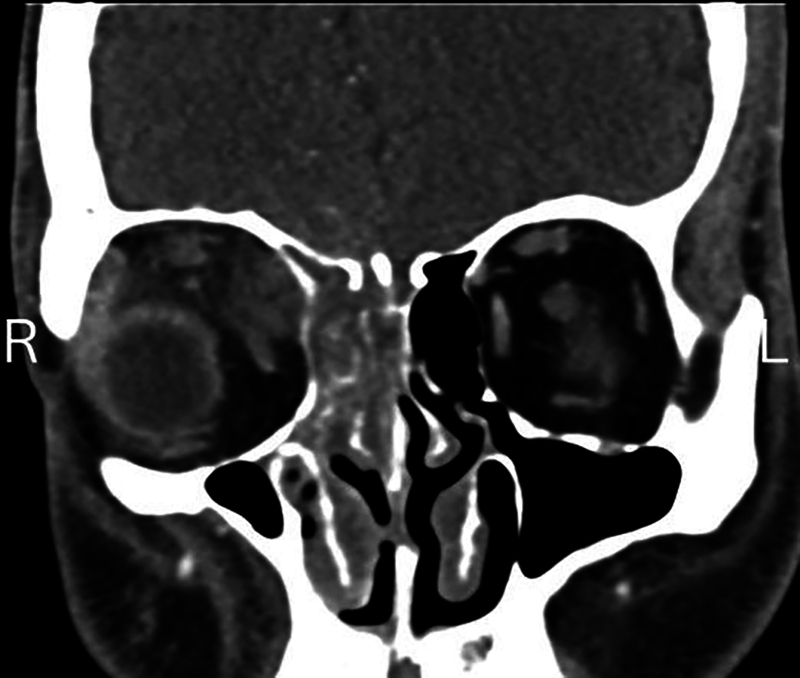
Preoperative coronal CT showing the sinonasal involvement of the infection.

A 29-year-old male with a history of DM diagnosed 18 months prior presented to the ED with altered mental status and dyspnea as a result of DKA. Imaging revealed extensive mucosal thickening of the right ethmoid, frontal, and sphenoid sinuses. Dehiscence through the right orbital wall and cribriform plate led to intracranial extension into the right frontal lobe, consistent with invasive fungal sinusitis ( Fig. 1 ).

The patient was started on intravenous vancomycin, piperacillin/tazobactam, and amphotericin B. Given the rapid progression and suspicion of mucormycosis, he was transferred for surgical intervention. Upon arrival, the patient exhibited right proptosis, V2 hypoesthesia, motility deficits, and vision of 20/40 in the right eye.

Pathological examination revealed hallmark features of mucormycosis, including vascular invasion and bone invasion, confirmed through special staining with Grocott's Methenamine Silver (GMS) and Periodic Acid-Schiff (PAS). Extensive coagulative necrosis and marked active chronic inflammation were identified in multiple tissues, including the inferior turbinate, posterior turbinate, and sinus contents. These findings suggest the angioinvasive nature of mucormycosis, with evidence of fungal hyphae infiltrating blood vessels and bone, contributing to ischemia and necrosis.

An expanded endonasal approach was performed to debride the anterior skull base fungal infection. Intraoperative pathology confirmed mucormycosis. Despite the surgery, the patient's vision deteriorated the following day from 20/40 to 20/100 with the development of an afferent pupillary defect (APD), indicating optic nerve compromise.

**Fig. 2:**
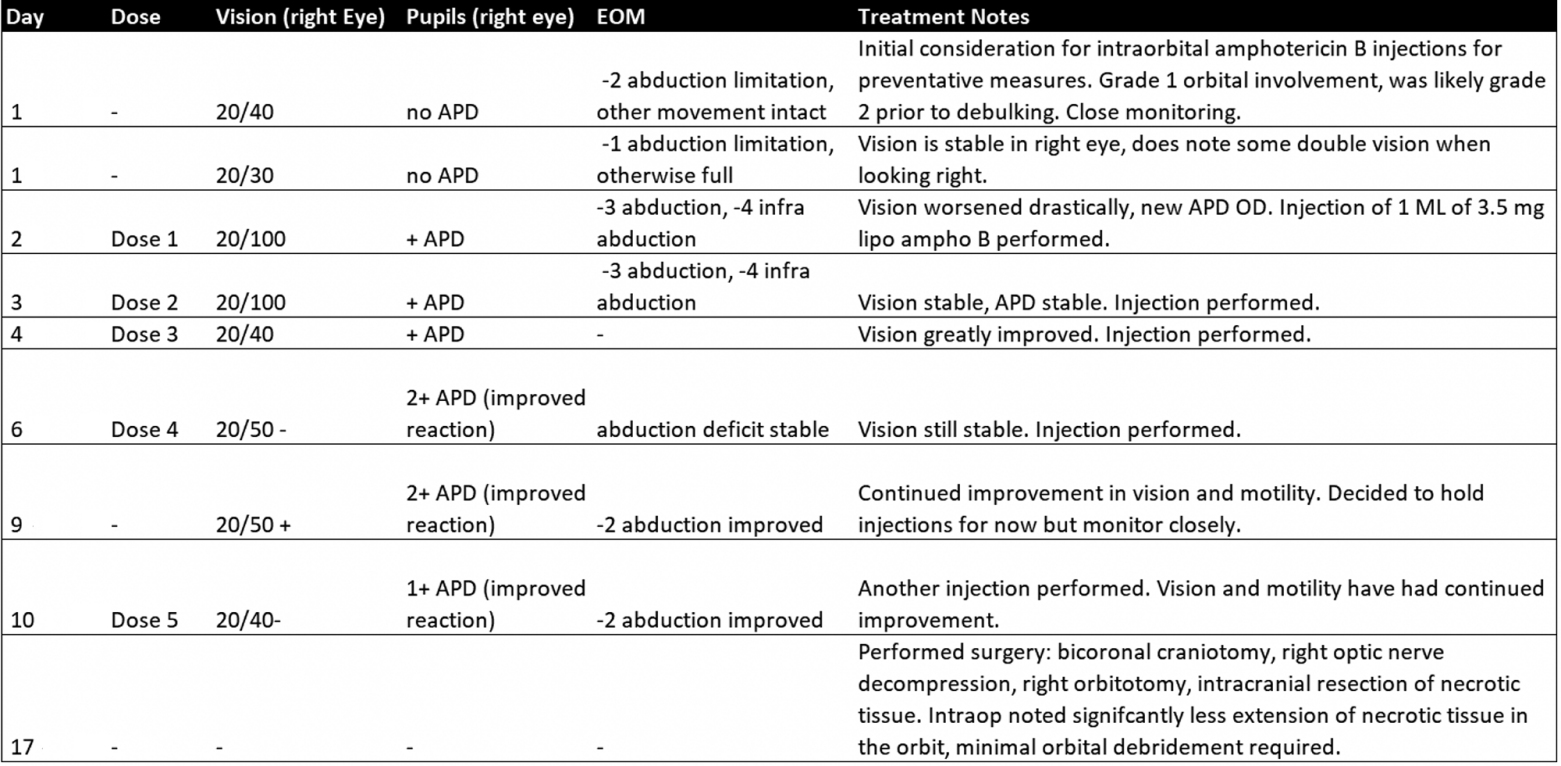
Table of the treatment course of 1 mL of 3.5 mg liposomal amphotericin B injections performed in the supramedial orbit. The table documents clinical improvements of vision from the injections. Abbreviations: APD, afferent pupillary defect; EOM, extraocular motility.

**Fig. 3:**
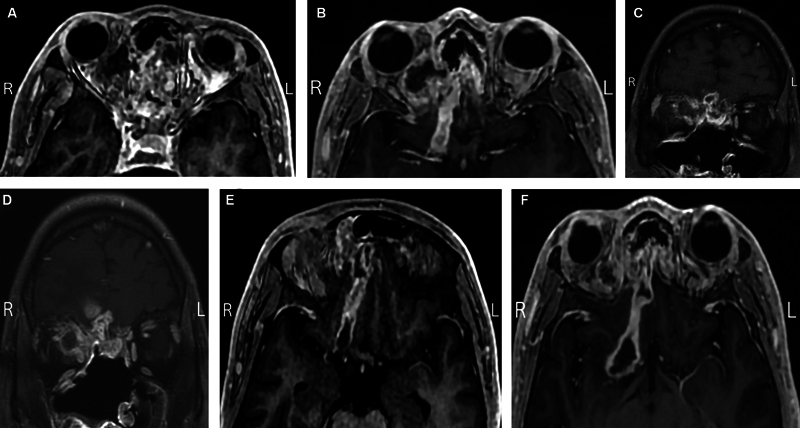
( **A, B** ) Axial MRIs taken before and after the liposomal amphotericin B injections, showing the orbital improvements. ( **C, D** ) Coronal MRIs taken before and after the liposomal amphotericin B injections further highlight the orbital improvement. ( **E, F** ) Axial MRIs taken before and after the liposomal amphotericin B injections, showing the intracranial progression.

Given the patient's reluctance to undergo further surgery, TRAMB therapy was initiated. The patient received 1 mL of 3.5 mg liposomal amphotericin B injections in the supramedial orbit. Visual acuity in the right eye improved to 20/40 by the third injection and the APD and abduction limitations were less pronounced ( Fig. 2 ). By the fifth injection, these symptoms remained stable ( Fig. 2 ). However, despite these orbital improvements, intracranial progression was noted subsequent MRIs ( Fig. 3A–F ).

**Fig. 4:**
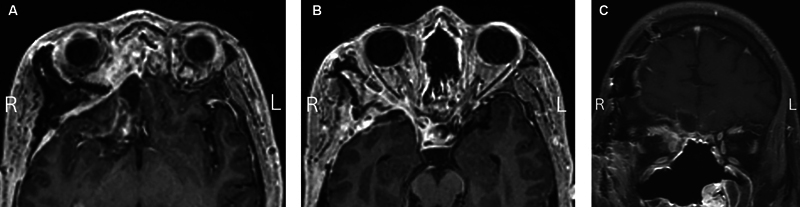
( **A** ) Postoperative axial MRI showing the intracranial cuts where the fungus was resected. **B** ) Postoperative axial MRI showing decompressed optic nerve and drastic decrease in proptosis. ( **C** ) Postoperative coronal MRI showing decompressed optic nerve.

The patient agreed to surgery, which was performed 1 week after his last injection. He underwent a bicoronal craniotomy, right optic nerve decompression, right orbitotomy, resection of necrotic tissue, and titanium mesh orbital wall reconstruction. Intraoperatively, we noted less extension of necrotic tissue in the orbit, requiring minimal orbital debridement. The surgery was successful in decompressing the optic nerve and addressing the intracranial invasion of the fungus ( Fig. 4A–C ).

## Postoperative Course

Following the surgical intervention, the patient exhibited gradual improvements in his vision. Intravenous amphotericin B was continued, and serial ophthalmological assessments confirmed stabilization of vision and resolution of the APD. A nasal endoscopy performed during follow-up revealed no cerebrospinal fluid leak and resolution of fungal disease. Postoperative imaging demonstrated a significant reduction in fungal burden with decreased proptosis ( Fig. 4 ).

## Discussion

The management of ROCM has historically relied on aggressive debridement and, in many cases, orbital exenteration for control of the fungal infection. 2 6 7 8 9 10 However, recent literature has demonstrated that TRAMB can stabilize orbital disease, serving as a valuable adjunct to debridement while avoiding the need for exenteration. 2 7 8 9 10 11 12 Sharifi et al. (2022) particularly found globe salvage rates of 95% and reductions in ophthalmic signs and symptoms like pain, proptosis, and chemosis with minimal side effects. 2

In previous studies, TRAMB was also used in order to delay progression in mucormycosis patients who needed surgical intervention until appropriate surgical intervention could be performed. 7 In our case, the decision to proceed with preoperative TRAMB was prompted by the patient's reluctance to undergo additional surgery despite the progression of orbital symptoms. In the delay period before he agreed to surgery, the injections improved the patient's vision and reduced the orbital fungal load, avoiding the need for orbital exenteration ( Fig. 3A–D ). Despite the positive orbital outcome, the patient's intracranial disease progressed ( Fig. 3E, F ).

Attempts have been made to use liposomal amphotericin B in other locations, such as the sinuses, with little benefit. 15 16 17 This is likely due to poor drug penetration into necrotic tissue and compromised blood flow caused by fungal progression in the sinuses. 15 Tissue necrosis primarily occurs from fungal hyphae invading blood vessels, causing thrombosis, ischemia, and subsequent tissue death. 4 The sinuses are generally more prone to fungal invasion and necrosis due to their thinner bony walls, limited blood supply, and close proximity to air-filled spaces, all of which are further exacerbated in immunocompromised patients. 18 In contrast, the orbital injections have demonstrated high effectiveness in treating the infected regions. This is likely due to the rich vascular supply and orbital fat. Further research is warranted to confirm this hypothesis.

Liposomal amphotericin B enhances drug distribution by encapsulating amphotericin B in lipid vesicles, improving its solubility and allowing for controlled, sustained release. 13 14 19 This formulation minimizes systemic toxicity by selectively releasing the drug in infected or damaged tissues with compromised vessels, which are characteristic of fungal infections. This targeted delivery mechanism allows for effective drug concentrations at the site of infection while reducing off-target effects. 19

Systemic antifungal therapy and surgical debridement remain the standard of care. However, our study supports findings that preoperative TRAMB can stabilize the orbit and reduce surgical complexity, serving as both an adjunct to debridement and an alternative to exenteration, even with intracranial disease progression.

## Conclusion

Preoperative administration of TRAMB, in this case, played a crucial role in stabilizing the patient's condition, preserving vision, and avoiding the need for immediate exenteration despite continued disease progression in the intracranial region. TRAMB is a promising, minimally invasive treatment option for patients with orbital involvement of mucormycosis who cannot undergo urgent surgery. Mechanistically, its effectiveness likely stems from the direct delivery of antifungal therapy to the well-vascularized orbital tissues. TRAMB is a safe and effective adjunct in the management of ROCM, particularly when surgery is delayed, offering an option for globe preservation while reducing the morbidity associated with more aggressive surgical procedures.
